# Polyphosphoester-Based Nanocarriers for Combined X-Ray-Induced Photodynamic Therapy and Immunotherapy

**DOI:** 10.3390/pharmaceutics18040399

**Published:** 2026-03-24

**Authors:** Han Zhang, Weijie Hu, Busharemu Reheman, Ningnannan Zhang, Junping Wang, Zhang Zhang, Chunyang Sun

**Affiliations:** Department of Radiology, Tianjin Key Laboratory of Functional Imaging & Tianjin Institute of Radiology, Tianjin Medical University General Hospital, Tianjin 300052, China

**Keywords:** polyphosphoesters, nanocarriers, X-ray-induced photodynamic therapy, immunotherapy, combination therapy

## Abstract

**Background**: The combination of photodynamic therapy (PDT) and immunotherapy has been explored as an innovative approach to enhance efficacy against tumors. However, PDT shows limited effectiveness in treating deep-seated tumors, as light and lasers do not sufficiently penetrate tissue. **Methods**: Herein, we introduced a nanocarrier (NP_VR_) via self-assembly, using an amphiphilic copolymer to co-deliver the hydrophobic photosensitizer verteporfin (VP) and the immunoadjuvant imiquimod (R837). **Results**: Our X-ray-induced photodynamic therapy (X-PDT) mechanism induced NP_VR_ to generate a large amount of cytotoxic reactive oxygen species (ROS), which directly killed cancer cells. Moreover, the released R837 facilitated immunogenic cell death following the X-PDT process and promoted the maturation of dendritic cells (DCs), thereby eliciting immune responses against malignant triple-negative breast cancer (TNBC). In animal experiments, the combined therapy using NP_VR_ showed a tumor growth inhibition rate of ~70%. **Conclusions**: This novel strategy opens new avenues to designing next-generation nanomedicines for use in immunotherapy and other combination therapies.

## 1. Introduction

Breast cancer is a multifaceted and complex condition marked by the unregulated proliferation of abnormal cells [1,2]. It is one of the most prevalent cancers globally and therefore significantly impacts public health [3]. The disease can arise in various regions of the breast, including the ducts and lobules, and can be classified into distinct subtypes based on its molecular features, such as hormone receptor status (e.g., estrogen and progesterone receptors) and HER2 expression [4,5]. Surgery remains the most common treatment option, including lumpectomy (tumor removal) or mastectomy (complete breast removal) [6,7]. In certain cases, the lymph nodes are also excised to determine whether the cancer has metastasized. Radiotherapy is commonly applied following surgery to eliminate remaining cancer cells and lower the likelihood of recurrence [8]. Chemotherapy may also be utilized pre- (neoadjuvant) or postoperatively (adjuvant) to shrink tumors or target remaining cancer cells, particularly for more aggressive types of breast cancer, such as TNBC [9]. This variant is characterized by an absence of HER2 expression and estrogen and progesterone receptors, rendering it less responsive to hormonal therapies and targeted treatments and often leading to a poorer prognosis [10,11,12].

In photodynamic therapy (PDT), a photosensitizer (a light-sensitive compound) is administered to the patient [13,14]. Once the photosensitizer has accumulated in the target tissue, typically tumors, the compound is exposed to a specific wavelength of light, usually in the red or near-infrared spectrum. This induces a photosensitization reaction that generates ROS such as singlet oxygen, resulting in the destruction of the targeted cells through damage to their components [15,16]. PDT has proven effective in treating superficial or localized tumors, particularly those on the skin, and is especially useful for cases that would be challenging to treat with surgery or radiation [17]. Despite the clinical benefits of this therapy, its wider use is hindered by the restricted penetration of light into tissues, especially for tumors located deep within the body [18]. To address these limitations, X-PDT has been developed as an improvement over traditional PDT [19,20,21]. In this approach, photosensitizers are activated by X-rays, allowing for the treatment of deep tumors that cannot be reached by conventional PDT due to the shallow penetration depth of UV or visible light [22]. To date, numerous studies have demonstrated that rare-earth-doped or semiconductor nanoparticles, known as nanoscintillators, are suitable for X-PDT [23,24].

Immunotherapy is a novel strategy that harnesses the body’s immune defenses to detect and destroy malignant cells [25]. Unlike traditional treatments such as surgery, chemotherapy, and radiation, immunotherapy does not directly target cancer cells but instead enhances the immune response to combat the tumor [26,27]. When combined with X-PDT, immunotherapy has the potential to further amplify the immune response by stimulating both the innate and adaptive immune systems, resulting in more comprehensive tumor control. While X-PDT primarily focuses on tumor ablation, immunotherapy can induce a systemic immune response, thereby reducing the risk of tumor recurrence and metastasis. Imiquimod (R837), a synthetic TLR7 agonist, is a typical immunoadjuvant that has demonstrated significant potential in cancer treatment [28,29]. Toll-like receptors (TLRs) are essential elements of the innate immune response, serving a pivotal function in detecting pathogen-associated molecular patterns (PAMPs) [30]. Specifically, TLR7 is activated by single-stranded RNA, a signature of many viral infections, as well as synthetic analogs like R837 [31,32]. Accordingly, combining R837 with X-PDT may have synergistic effects, where local tumor destruction via X-PDT generates tumor-associated antigens and activates the immune system, while R837 enhances the immune response, leading to systemic antitumor immunity.

**Scheme 1:**
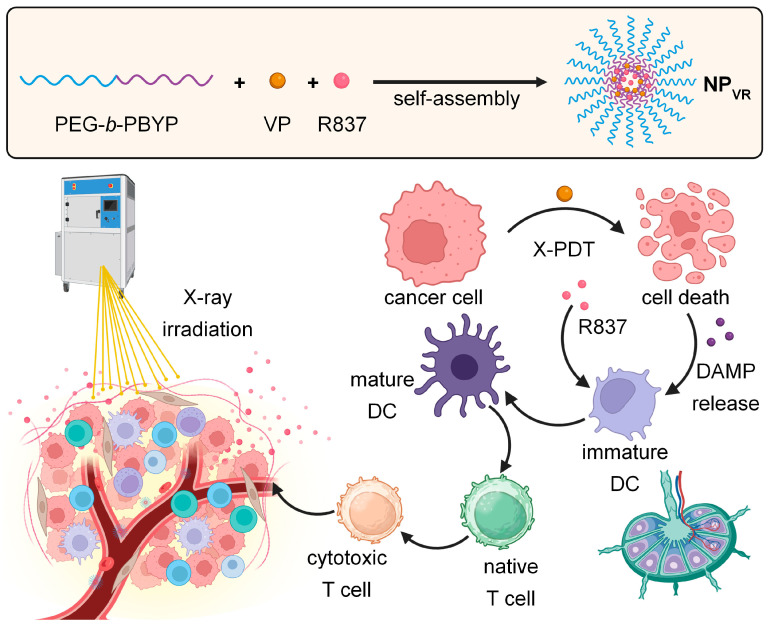
Preparation of the encapsulated VP and R837 nanomedicine (NP_VR_) and its application in combined X-PDT and cascaded immunotherapy.

Many photosensitizers and immunoadjuvants are hydrophobic, which often results in non-specific interactions with normal tissues and cells [33]. This impedes their targeting to tumor sites and contributes to substantial biocompatibility concerns in vivo. The recent emergence of nanomedicine is a significant breakthrough in the field of drug delivery, addressing various challenges associated with these agents [34,35]. Polyphosphoester is a biodegradable polymer with repetitive phosphoester linkages, allowing it to readily functionalize with various side groups. Recent research has highlighted its promising biocompatibility, generating considerable interest in the design of nanocarriers for cancer therapy [36,37,38]. Herein, we synthesized a diblock copolymer, PEG-*b*-PBYP, to enable the effective co-loading of verteporfin (VP, a photosensitizer) and R837 (an immunoadjuvant) and effectively combine X-PDT and cascaded immunotherapy (Scheme 1). After tail vein administration, the PEG modification of the nanocarrier (NP_VR_) shielded the encapsulated agents from being rapidly eliminated in the circulation and facilitated their preferential localization at tumor regions through passive accumulation pathways. Upon cellular uptake by cancer cells, exposure to X-rays in the presence of VP triggered the X-PDT process, producing ROS that induced cell death. Furthermore, R837 cooperated with the tumor-associated antigens released from damaged cells to enhance the maturation of DCs, stimulating a potent immune response. The combined therapeutic effects of NP_VR_ and X-ray exposure were thoroughly examined at both the cellular and animal levels.

## 2. Materials and Methods

### 2.1. Animals

Female BALB/c mice, aged six weeks, were obtained from HFK Bioscience Co., Ltd. (Beijing, China). All animal experiments adhered to the ethical protocols established by the Institute of Radiation Medicine under the Chinese Academy of Medical Sciences, with formal approval granted by its Institutional Animal Care and Use Committee (No. IRM-DWLL-2021031).

### 2.2. Preparation and Characterization of NP_VR_

The nanoprecipitation method was employed to fabricate NP_VR_ encapsulating VP and R837. Briefly, 500.0 μg of PEG-*b*-PBYP was mixed with 30 μL of dimethyl sulfoxide. VP and R837 were mixed in dimethyl sulfoxide, with a terminal concentration of 2.5 μg/μL. Following mixing, the obtained solution was added dropwise to 500 μL of deionized water while stirring gently. After 0.5 h of stirring, DMSO, along with any free VP and R837, was first removed through dialysis (using a membrane with a molecular weight cutoff of 8000–14,000 Da) against deionized water at 4 °C. Then, the solution was centrifuged (1000 rpm, 5 min) to further remove residual impurities. In a similar manner, micelles containing either VP or R837 were prepared using the same procedure and denoted using NP_V_ or NP_R_, respectively.

### 2.3. R837 Release from NP_VR_

To evaluate the in vitro release behavior of VP, suspensions of NP_R_, or NP_VR_ containing 500 μg of R837, were transferred into dialysis bags with a molecular weight cutoff of 8000–14,000 Da. These bags were submerged in phosphate buffer (PB) at neutral pH. The samples were incubated at physiological temperature with gentle shaking (80 rpm) and protected from light. At predetermined time points, aliquots of external medium were withdrawn and replaced with fresh PB. The concentration of R837 in the external medium was quantified using HPLC.

### 2.4. Cellular Uptake of NP_VR_

Murine breast cancer 4T1 cells were incubated with DMEM medium containing 10% FBS at a density of 2 × 10^5^ cells per well for 12 h at 37 °C. NP_V_ or NP_VR_ were incubated with 4T1 cells for 2, 6, and 12 h, and cellular uptake was then visualized using confocal microscopy. Meanwhile, NP_V_ or NP_VR_ were also incubated with 4T1 cells for different time periods, and then washed with chilled phosphate-buffered saline (PBS) and subsequently harvested. Following cell disruption, the amount of intracellular VP was quantified using a fluorescence spectrophotometer.

### 2.5. Combined Effect of NP_VR_ upon X-Ray Irradiation Against Bone Marrow-Derived Dendritic Cells (BMDCs)

4T1 cells were seeded into a 96-well plate at a density of 1 × 10^4^ cells per well and incubated with NP_V_, NP_R_, or NP_VR_. After 6 h, the medium was discarded, and blank medium was added. The cells were incubated for an additional 18 h and then irradiated by X-rays at 4 Gy for 10 min. Following an additional 48 h of incubation, HMGB1 expression and ATP secretion were measured using ELISA.

Bone marrow cells were extracted from the femurs of mice and subjected to centrifugation (500× *g*) and red blood cell lysis. The cells were then cultured in 6-well plates, and differentiation into bone marrow-derived dendritic cells (BMDCs) was induced with 20% FBS, GM-CSF, and IL-4. BMDCs were collected by harvesting both non-adherent and loosely adherent cells. These BMDCs were seeded and incubated with 4T1 cell supernatant, as described above, for 48 h. After incubation, IL-12 and TNF-α levels were measured using an ELISA assay.

### 2.6. Blood Clearance and Tumor Targeting of NP_VR_ In Vivo

Female BALB/c mice were randomly divided into three groups, each consisting of four mice. The mice received intravenous injections of either free VP, NP_V_, or NP_VR_ (equivalent [VP] = 10 mg per kilogram of body weight). Plasma samples (0.1 mL) were collected from the retro-orbital plexus at various time points: 10, 30, and 60 min, and 2, 4, 8, 12, 24, 48, and 72 h, post-injection. The photosensitizer content in the peripheral blood was then analyzed using a fluorescence spectrophotometer.

## 3. Results

### 3.1. Fabrication of Nanomedicine

**Figure 1:**
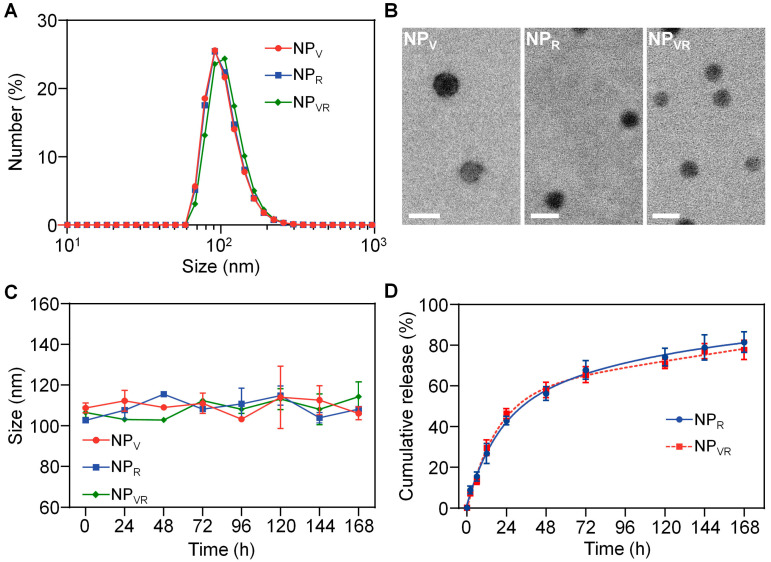
(**A**) DLS measurement of drug-loaded NP_V_, NP_R_, and NP_VR_. (**B**) TEM images of various polyphosphoester-based nanomedicines. Scale bar: 0.1 μm. (**C**) Stability of nanomedicines after incubation in PB at pH 7.4 for 7 days. (**D**) Cumulative R837 release behavior from NP_R_ and NP_VR_ for 168 h.

Following the preparation process, dynamic light scattering (DLS) analysis revealed that the average hydrodynamic sizes of NP_V_, NP_R_, and NP_VR_ were approximately 110 nm (Figure 1A), while their PDI values were 0.146, 0.111, and 0.112, respectively. As shown in Figure 1B, transmission electron microscopy (TEM) images demonstrated that NP_VR_ exhibited a typical micellar structure, characterized by spherical and compact cores, each measuring around 100 nm in diameter. Their surface potential was measured at −15.8, −17.2, and −14.5 mV. The drug loading content values of NP_VR_ were 2.81% for VP and 3.07% for R837, respectively, while the encapsulation efficiency of VP and R837 were 26.13% and 28.55%, respectively. Moreover, the incubation of NP_VR_ in DMEM containing 10% FBS at physiological temperature for one week did not result in any remarkable changes in diameter (Figure 1C). Next, the release patterns of R837 from NP_R_ and NP_VR_ under neutral conditions were examined. As displayed in Figure 1D, at pH 7.4, >77% of the total R837 was released from both NP_R_ and NP_VR_ after 7 days.

The production of ROS by NP_VR_ during the X-PDT process was initially investigated using DCFH-DA as a fluorescent probe. As shown in Figure S1A, both VP-loaded NP_V_ and NP_VR_ produced significant ROS upon exposure to 4 Gy of X-ray irradiation, a level comparable to that generated by free VP. To differentiate singlet oxygen (^1^O_2_) from other reactive species, 1,3-diphenylisobenzofuran (DPBF) was employed to monitor ^1^O_2_ production. Following X-ray exposure, DPBF absorption significantly decreased in both the NPV and NPVR groups, indicating the generation of ^1^O_2_ (Figure S1B). Moreover, the addition of L-histidine, a ^1^O_2_ quencher, notably reduced the rate of DPBF absorption decay. The content of superoxide anions (O_2_^−^) and hydroxyl radicals (·OH) during X-PDT was also measured using dihydrorhodamine 123 and hydroxyphenyl fluorescein, respectively (Figures S1C,D). These findings demonstrate that NP_VR_ can generate cytotoxic ROS capable of potentially killing cancer cells.

### 3.2. Internalization of NP_VR_ and Effect of X-PDT at the Cellular Level

**Figure 2:**
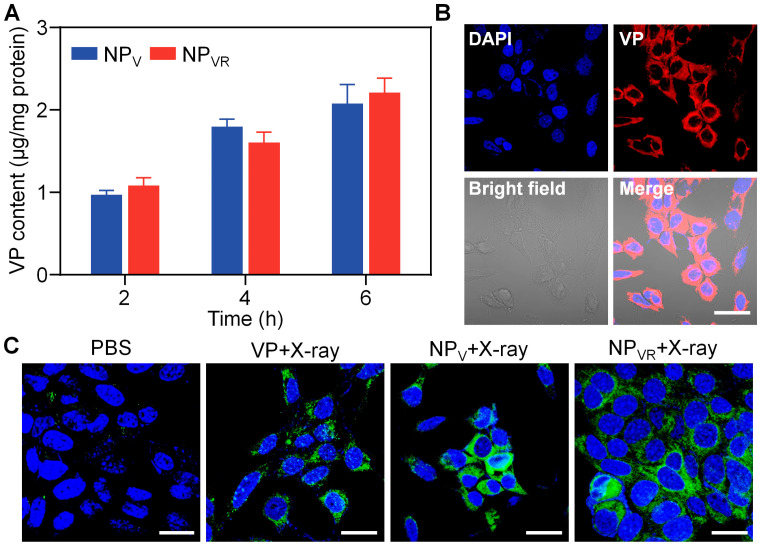
(**A**) Intracellular VP content following incubation with NP_V_ or NP_VR_ for 2, 6, and 12 h. (**B**) Cellular uptake of NP_VR_ for 2 h observed using confocal microscopy. The scale bar is 30 μm. (**C**) Intracellular ROS production of 4T1 cells treated with different formulations upon X-ray exposure. The scale bar is 20 μm.

We then evaluated the cellular internalization of NP_VR_ by quantitatively measuring the intracellular levels of VR. 4T1 cells were exposed to free VP, NP_V_, or NP_VR_ for varying time intervals, and internalized VP was quantified using fluorescence spectroscopy. As shown in Figure 2A, longer incubation times led to intracellular VP content gradually increasing in 4T1 cells incubated with either NP_V_ or NP_VR_. After 12 h of incubation, NP_VR_ facilitated the intracellular uptake of VP, with values of 2.21 ± 0.18 μg/mg protein achieved. The internalized NP_VR_ was also visualized using confocal microscopy. As expected, remarkable VP signals were detected in the cytoplasm after incubating 4T1 cells with NP_VR_ for 6 h (Figure 2B), indicating efficient cellular internalization. Subsequently, CLSM was employed using specific fluorescent probes to investigate NP_VR_-triggered ROS production during X-PDT. Following X-ray exposure, both NP_V_ and NP_VR_ exhibited strong intracellular ROS signals (Figure 2C). It was worth noting that similar levels of ROS were observed in both groups, attributed to the comparable internalization behavior of VP.

### 3.3. Combined Effect of NP_VR_ Under X-Ray Irradiation at the Cellular Level

**Figure 3:**
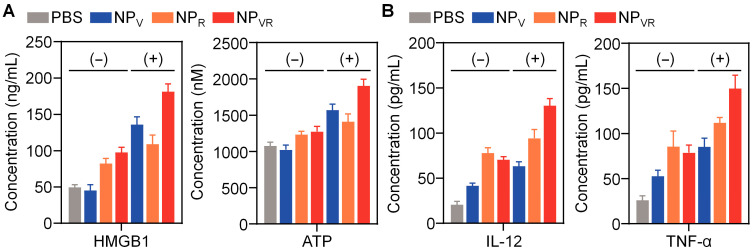
(**A**) HMGB1 and ATP content of 4T1 cells incubated with various formulations. (**B**) TNF-α and IL-12 content in supernatant of DCs after treatment with various formulations.

Previous studies have shown that PDT can trigger ICD by promoting the expression of various DAMPs, which subsequently lead to productive immune responses [39,40]. To investigate this, we assessed the expression of HMGB1, as well as ATP secretion, in 4T1 cells following various treatments. Compared to the other groups, clear elevations in extracellular HMGB1 and ATP were found in 4T1 cells treated with NP_V_+X-ray or NP_VR_+X-ray (Figure 3A). Based on these results, we next studied the role of R837 as an immunoadjuvant to stimulate immune responses. As shown in Figure 3B, the highest production of related cytokines, such as TNF-α and IL-12, was observed in the NP_VR_+X-ray group, further confirming the presence of boosted immune responses. Regardless of X-ray irradiation, the blank NP did not induce significant immune activation (Figure S2), indicating the negligible intrinsic effect of the nanocarrier itself. This further confirms that the therapeutic/immunological effects observed for NP_VR_ primarily originated from the encapsulated drug molecules (VP and R837), rather than the NP nanoassembly.

### 3.4. Pharmacokinetics and Biodistribution of NP_VR_ at the Animal Level

**Figure 4:**
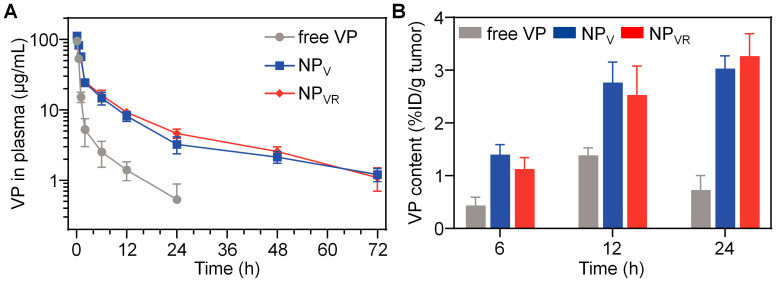
(**A**) VP concentration in plasma over time following systemic administration of free VP, NP_V_, or NP_VR_ (*n* = 4, mean ± SD). (**B**) VP content in tumor sites 6, 12, and 24 h after administration of various formulations.

The pharmacokinetics of the PEGylated nanocarriers were studied in female BALB/c mice (*n* = 4). Blood samples were taken at various time points (0.083, 0.5, 1, 2, 6, 12, 24, 48, and 72 h after injection) to determine the VP content. Both NP_V_ and NP_VR_ exhibited prolonged circulation in the peripheral blood, whereas free VP was rapidly cleared after intravenous administration (Figure 4A). The plasma content of VP in mice treated with free VP was measured at 0.52 ± 0.35% of the total administered dose 24 h after administration. However, the circulation of VP was extended by NP_V_ and NP_VR_, with plasma concentrations of 1.21 ± 0.25% (NP_V_) and 1.10 ± 0.41% (NP_VR_) of the initial dose, respectively, even at 72 h after administration. In addition, 4T1 tumor-bearing mice received systemic administration of free VP, NP_V_, or NP_VR_. At different time intervals, tumor tissues were harvested to determine VP quantification. As expected, 24 h after administration, the VP content in the free VP group was 0.72 ± 0.28% of ID per gram of tumor (Figure 4B). In comparison, the distribution of VP in the NP_V_ and NP_VR_ groups was measured as 3.03 ± 0.24% and 3.27 ± 0.42% of ID/g tumor at 24 h after administration, respectively, 4.21- and 4.54-fold higher than that in the control group.

### 3.5. NP_VR_-Induced Tumor Growth Inhibition Under X-Ray Irradiation at the Animal Level

**Figure 5:**
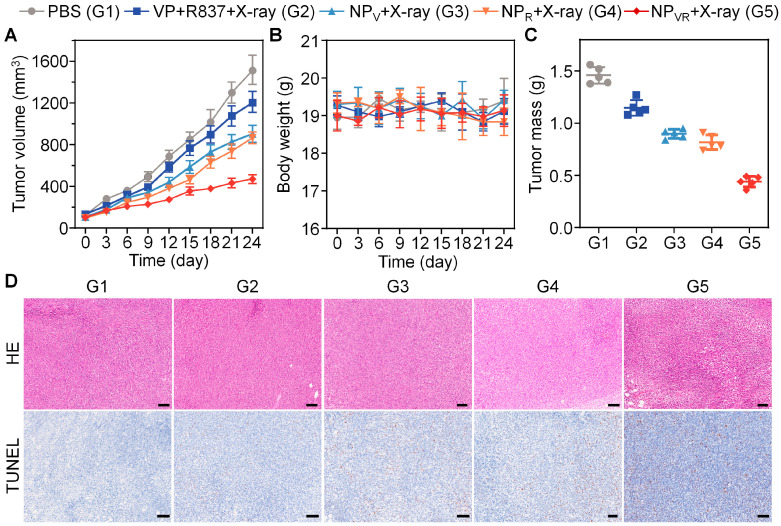
(**A**) Comparison of 4T1 tumor volume between groups during the entire therapeutic period. (**B**) Body weight comparison of 4T1 tumor-bearing mice receiving various treatments. (**C**) Mass of tumor tissues after treatment. (**D**) H&E and TUNEL staining of tumor sections from mice receiving various treatments. The scale bar is 100 μm.

4T1 xenograft-bearing mice (with a total of 1 × 10^6^ 4T1 cells implanted) were randomly assigned to five groups once the tumor size reached approximately 100 mm^3^. Each mouse received one of the following treatments depending on the group: PBS, VP+R837, NP_V_, NP_R_, or NP_VR_. Each treatment consisted of a systemic injection, administered once a week. Additionally, the mice in the VP+R837, NP_V_, NP_R_, and NP_VR_ groups were exposed to X-rays 24 h after each administration. Throughout the treatment period, changes in tumor size and body mass were recorded twice a week. Due to inevitable clearance and inadequate tumor distribution, no significant difference in tumor volume was observed between the PBS and VP+R837+X-ray groups (Figure 5A). By day 24, the average tumor size in the PBS control group exceeded 1510 mm^3^, whereas the NP_V_+X-ray treatment significantly delayed tumor growth, with a final tumor volume of 865 ± 57 mm^3^. Notably, the NP_VR_+X-ray group exhibited the most substantial antitumor effect, achieving a tumor inhibition rate (TIR) of ~70%, indicating marked growth inhibition rather than a curative outcome. However, despite the pronounced tumor suppression, complete tumor eradication was not achieved during the observation period. Importantly, the NP_VR_+X-ray treatment also significantly prolonged survival, with 60% of tumor-bearing mice remaining alive on day 60 (Figure S3). Meanwhile, mouse body weight remained relatively stable across all groups throughout the 24-day monitoring period, indicating that the NP_VR_+X-ray treatment was well-tolerated (Figure 5B). On day 25, tumors were dissected and weighed. As shown in Figure 5C, the NP_VR_+X-ray group showed the lowest tumor weight, further confirming the potent antitumor efficiency of this treatment. Histopathological analysis of the tumor tissue (Figure 5D) revealed an increase in TUNEL expression in the NP_VR_+X-ray group.

**Figure 6:**
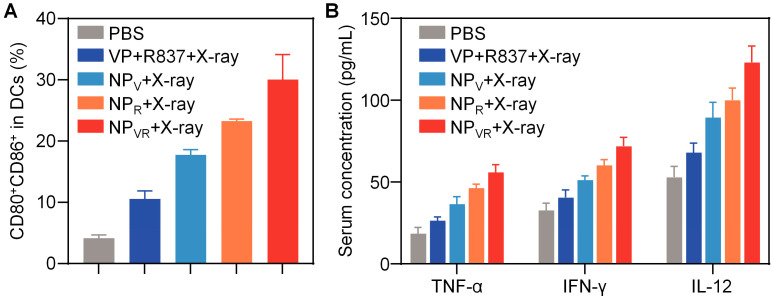
(**A**) DC maturation in the tumor-draining lymph nodes of mice treated with different formulations. (**B**) TNF-α, INF-γ, and IL-12 content in serum of mice treated with different formulations.

To evaluate the potential of the NP_VR_+X-ray treatment in enhancing DC maturation, draining lymph nodes were examined using flow cytometry. As anticipated, the NP_VR_+X-ray group demonstrated a markedly elevated DC maturation rate of 30.03 ± 4.07%, significantly surpassing the levels observed in the PBS, VP+R837+X-ray and NP_V_+X-ray groups (Figure 6A). More importantly, X-ray irradiation in combination with NP_VR_ further increased the proportion of intratumoral CD8^+^ T cells (Figure S4). Pro-inflammatory cytokines such as TNF-α, IFN-γ, and IL-12 exhibited a general uptrend in mouse serum after treatment with various nanoparticulate formulations upon X-ray irradiation, with the NP_VR_+X-ray group showing the most pronounced cytokine production (Figure 6B). Importantly, the blank NP carrier did not exhibit any measurable tumor-inhibitory effect in vivo (Figure S5). Additionally, the serum levels of these cytokines in the NP-treated group were similar to those in the PBS group (Figure S6), suggesting that the enhanced therapeutic efficacy of the NP_VR_+X-ray treatment is primarily due to the loaded therapeutic agents and their action under X-ray irradiation, rather than any intrinsic effect of the nanoparticle carrier itself.

To evaluate long-term immune memory beyond the antitumor effect at the primary tumor, a distant tumor rechallenge model was established to simulate tumor relapse (Figure 6A). As effector memory T (T_EM_) cells are critical for protection against recurrence, CD4^+^CD62L^low^CD44^high^ T_EM_ cells in the spleen were quantified. The NP_VR_+X-ray group showed the highest T_EM_ proportion (53.23 ± 2.72%), which was 1.87-, 1.60-, 1.37-, and 1.16-fold higher than those of the PBS, VP+R837+X-ray, NP_V_+X-ray, and NP_R_+X-ray groups, respectively (Figure S7A). Consistently, after contralateral tumor rechallenge, mice pretreated with the NP_VR_+X-ray regime exhibited significantly slower secondary tumor growth than those in the PBS group (Figure S7B). These findings suggest that the NP_VR_+X-ray treatment markedly inhibits 4T1 tumor relapse through the induction of systemic immune memory.

### 3.6. Biocompatibility Assessment of NP_VR_ at the Animal Level

**Figure 7:**
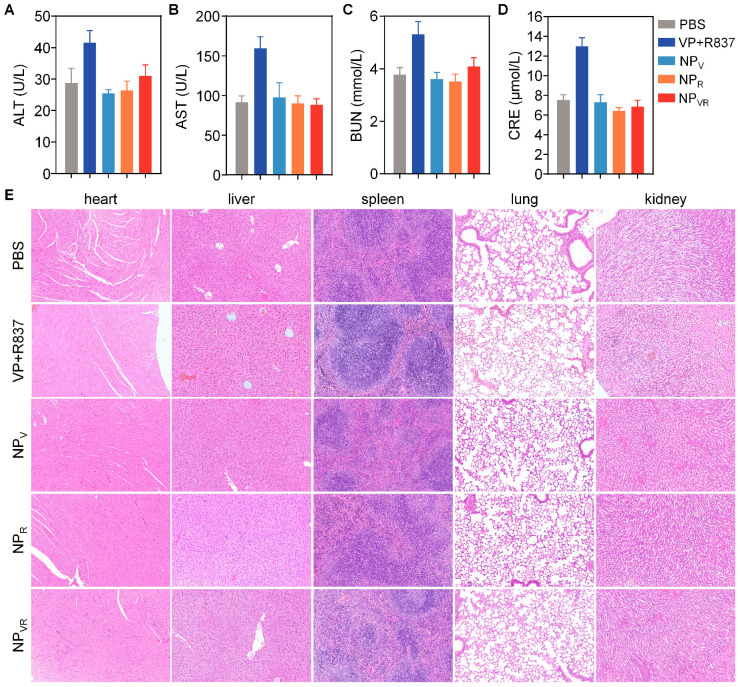
(**A**–**D**) Plasma levels of blood biochemical indicators in BALB/c mice after intravenous administration of different formulations. (**E**) Histopathological H&E staining of various tissues of mice treated with different formulations. Magnification: 20×.

To assess the in vivo biocompatibility of our developed nanoparticulate drug delivery system (NP_VR_), female BALB/c mice were intravenously administered with PBS, free VP&R837, NP_V_, NP_R_, or NP_VR_ via the tail vein, and their body weight was monitored continuously for one week. As shown in Figure S8, the mice treated with NP_V_, NP_R_, or NP_VR_ exhibited minimal body weight fluctuations, illustrating that nanocarrier administration caused negligible systemic toxicity. When compared to the VP+R837 group, negligible variations in frequently used parameters were found for the control and NP_V_-, NP_R_-, and NP_VR_-treated groups (Figure 7A–D). As shown in Figure 7E, our histopathological analysis demonstrates the satisfactory biocompatibility of NP_VR_ and highlights a significant reduction in toxicity upon the co-loading of both therapeutic agents.

## 4. Discussion

As Wooley et al., among other researchers, have reported on the hydrophobic nature of the PBYP domain, we fabricated the corresponding nanocarriers (termed NP_VR_) using amphiphilic PEG-*b*-PBYP and a simple nanoprecipitation method driven by hydrophobic–hydrophobic interactions. In parallel, nanocarriers loaded individually with either VP (termed NP_V_) or R837 (termed NP_R_) were prepared with a comparable strategy. The negative zeta potential and observed reduction in size from the DLS measurements compared to TEM imaging could be attributed to the presence of the PEG hydration layer. The PBYP segment facilitated highly efficient loading of both the hydrophobic photosensitizer and immunoadjuvant. The good level of stability demonstrated in a complete cell culture medium suggests that PEGylation provides protection and enhances the stability of nanocarriers in physiological environments. The sustained release of R837 from nanoparticulate cores is likely due to the degradation of polyphosphoesters, which enabled cascaded immunotherapy after the X-PDT treatment. Our results demonstrate that both agents, each with a small molecular weight, can be effectively loaded within PEG-*b*-PBYP, with R837 being rapidly released to act as an immunoadjuvant.

In the absence of a dedicated nanoscintillator, the X-PDT effect of VP in our system is more likely to be caused by radiation-induced photophysical and radiochemical processes, rather than a classical scintillator-mediated energy-transfer mechanism. Upon high-energy X-ray irradiation, the aqueous biological microenvironment undergoes radiolysis, generating short-lived reactive intermediates and energetic secondary electrons. Additionally, megavoltage X-ray exposure can induce Cherenkov radiation in tissue-like media. Since Cherenkov emissions contain a broad UV–visible spectral component that overlaps with the absorption profile of VP, they can serve as an internal optical source to promote VP excitation. Moreover, secondary electrons produced during irradiation may further facilitate ROS generation through electron-driven interactions with VP and surrounding molecular oxygen. Therefore, the X-PDT activity of VP observed in our study is likely mediated by the combined contributions of Cherenkov-assisted photoexcitation and secondary electron-amplified oxidative reactions, ultimately leading to enhanced intracellular ROS production and tumor cell damage. This interpretation aligns with previous reports demonstrating that VP-containing nanoconstructs can generate singlet oxygen and exert enhanced cytotoxicity under 6 MeV X-ray irradiation, even in the absence of a conventional nanoscintillator [41,42].

Due to the active mechanisms and specific cellular uptake sites of VP and R837, their internalization is crucial to ensuring theirs functions as a photosensitizer and an immunoadjuvant, respectively. The internalization of NP_VR_ was investigated using both CLSM and a fluorescence spectrophotometer. After X-ray irradiation at 4 Gy, significant ROS generation was confirmed using specific fluorescent probes. Our findings suggest that NP_VR_ may intensify intracellular oxidative stress, potentially promoting R837 activation for cascaded immunotherapy.

In our design, PEG chains grafted onto the surface of NP_VR_ reduce macrophage recognition within the circulatory system, thus extending the systemic circulation time of the payload. Unlike the free VP, which was rapidly eliminated, NP_VR_ demonstrated sustained cargo retention in the circulation and consequently enhanced the targeted distribution of VP in tumor tissues following administration.

Although this enhanced permeability and retention (EPR) effect may contribute to the accumulation of NP_VR_ in tumors, it should not be regarded as the sole mechanism responsible for the tumor localization observed in this study. Given the well-documented heterogeneity and limited clinical predictability of the EPR effect, the in vivo enrichment of our formulation is more likely attributable to the combined influence of passive nanoscale distribution, prolonged circulation, reduced systemic clearance, and surface PEGylation features that may enhance tumor retention and uptake [34,43]. The prolonged circulation of both the photosensitizing agent and immunoadjuvant, coupled with their increased distribution in tumor sites, prompted further evaluation of NP_VR_’s therapeutic efficacy in a murine 4T1 breast cancer model. Tumor progression was tracked across the entire treatment period, and tumor weights were recorded post-therapy. Both measurements show that the NP_VR_+X-ray group demonstrated the highest tumor growth inhibition. It should be noted, however, that the in vivo therapeutic outcome observed in this study reflected significant tumor growth inhibition rather than complete tumor eradication. Moreover, our analysis of DC maturation, CD8^+^ T cell proportion, and inflammatory cytokine secretion illustrated the ability of the NP_VR_+X-ray treatment to elicit robust immune responses. A tumor rechallenge assay, together with our analysis of T_EM_ cells, further demonstrated that the treatment induced durable systemic immune memory capable of suppressing 4T1 tumor recurrence.

Previous studies have demonstrated that PEGylated polyphosphoester-based nanoparticles represent promising drug delivery platforms because their phosphoester linkages are susceptible to hydrolytic degradation. Owing to their phosphorus-containing backbone, these materials produce phosphate-based small molecules upon degradation, depending on the side-chain structure, which may confer superior biocompatibility relative to certain aliphatic polyesters that generate acidic degradation by-products. In the present study, PEGylated amphiphilic polyphosphoesters enabled the fabrication of self-assembled nanocarriers with favorable in vivo tolerability. Consistent with this material property, liver and kidney function analyses, together with H&E staining of major organs, supported the acceptable biosafety of the NP_VR_+X-ray treatment throughout the observation period. Collectively, these findings indicate that NPVR+X-ray effectively suppresses tumor growth through the combined actions of X-PDT and cascaded immunotherapy while maintaining a satisfactory biocompatibility profile.

## 5. Conclusions

In this study, a biocompatible nanomedicine was designed, based on hydrophobic polyphosphoesters, to simultaneously deliver VP and R837, enabling a combination of X-PDT and cascaded immunotherapy. Both our in vitro and in vivo results demonstrate that NP_VR_ effectively functions as both a photosensitizer and an immunoadjuvant in the treatment of malignant TNBC. The combined treatment regime led to a marked increase in anticancer efficacy when compared to either therapeutic modality individually, as shown by its improvements in cancer cell killing and immune responses. More importantly, irradiation with a low-dose X-ray alone did not result in noticeable cell death. In addition, the NP_VR_ formulation not only demonstrated remarkable inhibition of 4T1 tumor growth but also caused no significant toxicity in mice. Our study underscores the potential of polyphosphoester-based nanomedicines in combination therapies involving immunotherapy and highlights new opportunities for their broader application in the future, significantly contributing to their potential clinical use.

## Data Availability

The original contributions presented in this study are included in the article/Supplementary Material. Further inquiries can be directed to the corresponding authors.

## Supplementary Materials

The following supporting information can be downloaded at: https://www.mdpi.com/article/10.3390/pharmaceutics18040399/s1, Figure S1: (A) relative DCF fluorescence intensity at 525 nm in different groups. (B) UV–vis spectra of DPBF in different groups. (C) fluorescence spectra of dihydrorhodamine 123 in different groups. (D) Relative fluorescence intensity of hydroxyphenyl fluorescein at 515 nm in different groups. (+): X-ray irradiation.; Figure S2: HMGB1 (A) and ATP (B) content of 4T1 cells incubated with NP. IL-12 (C) and TNF-α (D) content in supernatant of DCs after treated with NP; Figure S3: survival of mice after treatment with various formulations; Figure S4: CD8^+^ T cells in tumors after the treatments; Figure S5: (A) 4T1 tumor volume in different groups during the whole therapeutic period. (B) Monitor of body weight of 4T1 tumor-bearing mice received treatment with PBS or NP. (C) Mass of tumor tissues after the treatment; Figure S6: TNF-α, INF-γ and IL-12 content in serum of mice treated with PBS or NP; Figure S7: (A) CD4^+^ memory T cells (CD3^+^CD4^+^CD44^high^CD62L^low^) in spleen of mice treated with various formulations. (b) Relative 4T1 tumor growth after the tumor rechallenges. Figure S8: Body weight change of mice after treatment with various formulations. Reference [44] is cited in supplementary materials.

## References

[1] Bray F., Laversanne M., Sung H., Ferlay J., Siegel R., Soerjomataram I., Jemal A. (2024). Global cancer statistics 2022: GLOBOCAN estimates of incidence and mortality worldwide for 36 cancers in 185 countries. CA-Cancer J. Clin..

[2] Modi S., Jacot W., Yamashita T., Sohn J., Vidal M., Tokunaga E., Tsurutani J., Ueno N.T., Prat A., Chae Y.S. (2022). Trastuzumab deruxtecan in previously treated HER2-low advanced breast cancer. N. Engl. J. Med..

[3] Wilkinson L., Gathani T. (2022). Understanding breast cancer as a global health concern. Br. J. Radiol..

[4] Zagami P., Carey L.A. (2022). Triple negative breast cancer: Pitfalls and progress. npj Breast Cancer.

[5] Victora C.G., Bahl R., Barros A.J.D., Franca G.V.A., Horton S., Krasevec J., Murch S., Sankar M.J., Walker N., Rollins N.C. (2016). Breastfeeding in the 21st century: Epidemiology, mechanisms, and lifelong effect. Lancet.

[6] Waks A.G., Winer E.P. (2019). Breast cancer treatment: A review. JAMA J. Am. Med. Assoc..

[7] Goldhirsch A., Winer E.P., Coates A.S., Gelber R.G., Piccart-Gebhart M., Thürlimann B., Panel members (2013). Personalizing the treatment of women with early breast cancer: Highlights of the St Gallen International Expert Consensus on the Primary Therapy of Early Breast Cancer 2013. Ann. Oncol..

[8] McLaughlin M., Patin E.C., Pedersen M., Wilkins A., Dillon M.T., Melcher A.A., Harrington K.J. (2020). Inflammatory microenvironment remodelling by tumour cells after radiotherapy. Nat. Rev. Cancer.

[9] Minckwitz G., Untch M., Blohmer J., Costa S.D., Eidtmann H., Fasching P.A., Gerber B., Eiermann W., Hilfrich J., Huober J. (2021). Definition and impact of pathologic complete response on prognosis after neoadjuvant chemotherapy in various intrinsic breast cancer subtypes. J. Clin. Oncol..

[10] Yin L., Duan J.J., Bian X.W., Yu S.C. (2020). Triple-negative breast cancer molecular subtyping and treatment progress. Breast Cancer Res..

[11] Bianchini G., Balko J.M., Mayer I.A., Sanders M.E., Gianni L. (2016). Triple-negative breast cancer: Challenges and opportunities of a heterogeneous disease. Nat. Rev. Clin. Oncol..

[12] Cameron D., Piccart-Gebhart M.J., Gelber R.D., Procter M., Goldhirsch A., de Azambuja E., Castro G., Untch M., Smith I., Gianni L. (2017). 11 years’ follow-up of trastuzumab after adjuvant chemotherapy in HER2-positive early breast cancer: Final analysis of the HERceptin Adjuvant (HERA) trial. Lancet.

[13] Li X.S., Lovell J.F., Yoon J., Chen X.Y. (2020). Clinical development and potential of photothermal and photodynamic therapies for cancer. Nat. Rev. Clin. Oncol..

[14] Kwiatkowski S., Knap B., Przystupski D., Saczko J., Kędzierska E., Knap-Czop K., Kotlińska J., Michel O., Kotowski K., Kulbacka J. (2018). Photodynamic therapy-mechanisms, photosensitizers and combinations. Biomed. Pharmacother..

[15] Pham T.C., Nguyen V., Choi Y., Lee S., Yoon J. (2021). Recent strategies to develop innovative photosensitizers for enhanced photodynamic therapy. Chem. Rev..

[16] Zhao X.Z., Liu J.P., Fan J.L., Chao H., Peng X.J. (2021). Recent progress in photosensitizers for overcoming the challenges of photodynamic therapy: From molecular design to application. Chem. Soc. Rev..

[17] Kaur R., Bhardwaj A., Gupta S. (2023). Cancer treatment therapies: Traditional to modern approaches to combat cancers. Mol. Biol. Rep..

[18] Guo C.Q., Lin L., Wang Y.H., Jing J., Gong Q.Y., Luo K. (2025). Nano drug delivery systems for advanced immune checkpoint blockade therapy. Theranostics.

[19] Yu Y.W., Liang L.S., Zhang X.W., Zhang L., Ni Z.Q., Zhu Z.H., Liu Y., Lan J., Liu W., Xie G. (2023). Pure organic AIE nanoscintillator for X-ray mediated type I and type II photodynamic therapy. Adv. Sci..

[20] Xie J.L., Wang Y.W., Choi W., Jangli P., Ge Y.Q., Xu Y.J., Kang J., Liu L., Zhang B., Xie Z. (2021). Overcoming barriers in photodynamic therapy harnessing nano-formulation strategies. Chem. Soc. Rev..

[21] Lu K.D., He C.B., Guo N.N., Chan C., Ni K.Y., Lan G.X., Tang H.D., Pelizzari C., Fu Y.-X., Spiotto M.T. (2018). Low-dose X-ray radiotherapy-radiodynamic therapy via nanoscale metal-organic frameworks enhances checkpoint blockade immunotherapy. Nat. Biomed. Eng..

[22] Wang X., Sun W.J., Shi H.F., Ma H.L., Niu G.W., Li Y.X., Zhi J.H., Yao X.K., Song Z.C., Chen L. (2022). Organic phosphorescent nanoscintillator for low-dose X-ray-induced photodynamic therapy. Nat. Commun..

[23] Sun W.J., Zhou Z.J., Pratx G., Chen X.Y., Chen H.M. (2020). Nanoscintillator-mediated X-ray induced photodynamic therapy for deep-seated tumors: From concept to biomedical applications. Theranostics.

[24] Chen H.M., Sun X.L., Wang G.D., Nagata K., Hao Z.L., Wang A., Li Z.B., Xie J., Shen B.Z. (2017). LiGa_5_O_8_:Cr-based theranostic nanoparticles for imaging-guided X-ray induced photodynamic therapy of deep-seated tumors. Mater. Horiz..

[25] Goodman A.M., Kato S., Bazhenova L., Patel S.P., Frampton G.M., Miller V., Stephens P.J., Daniels G.A., Kurzrock R. (2017). Tumor mutational burden as an independent predictor of response to immunotherapy in diverse cancers. Mol. Cancer Ther..

[26] Zugazagoiti J., Guedes C., Ponce S., Ferrer I., Molina-Pinelo S., Paz-Ares L. (2016). Current challenges in cancer treatment. Clin. Ther..

[27] Cheng A., Hsu C., Chan S.L., Choo S., Kudo M. (2020). Challenges of combination therapy with immune checkpoint inhibitors for hepatocellular carcinoma. J. Hepatol..

[28] Ni K.Y., Luo T.K., Nash G.T., Lin W.B. (2020). Nanoscale metal-organic frameworks for cancer immunotherapy. Acc. Chem. Res..

[29] Huang J., Leng X.J., Jiang T., Xu L.H., Zheng J., Fang M.X., Wang J., Wang Z., Zhang L. (2023). Oxygen-carrying nanoplatform to reprogram tumor immunosuppressive microenvironment and enhance photothermal-immunotherapy. Mater. Today Bio.

[30] Chen Q., Xu L.G., Liang C., Wang C., Peng R., Liu Z. (2016). Photothermal therapy with immune-adjuvant nanoparticles together with checkpoint blockade for effective cancer immunotherapy. Nat. Commun..

[31] van der Fits L., Mourits S., Voerman J.S.A., Kant M., Boon L., Laman J.D., Cornelissen F., Mus A.-M., Florencia E., Prens E.P. (2009). Imiquimod-induced psoriasis-like skin inflammation in mice is mediated via the IL-23/IL-17 axis. J. Immunol..

[32] Gardner A., Pulido A.d.M., Ruffell B. (2020). Dendritic cells and their role in immunotherapy. Front. Immunol..

[33] Shu G., Zhu W., Jiang X.Z., Li X.W., Pan J.B., Zhang X.N., Zhang X.J., Sun S.K. (2021). Persistent luminescence immune hydrogel for photodynamic-immunotherapy of tumors in vivo. Adv. Funct. Mater..

[34] Wilhelm S., Tavares A.J., Dai Q., Ohta S., Audet J., Dvorak H.F., Chan W.C.W. (2016). Analysis of nanoparticle delivery to tumours. Nat. Rev. Mater..

[35] Suk J.S., Xu Q.G., Kim N., Hanes J., Ensign L.M. (2016). PEGylation as a strategy for improving nanoparticle-based drug and gene delivery. Adv. Drug Deliv. Rev..

[36] Zhu Y.Q., Song Y.H., Cao Z.Y., Dong L., Lu Y., Yang X.Z., Wang J. (2021). Magnetically actuated active deep tumor penetration of deformable large nanocarriers for enhanced cancer therapy. Adv. Funct. Mater..

[37] Zhang F., Smolen J.A., Zhang S., Li R., Shah P.N., Cho S., Wang H., Raymond J.E., Cannon C.L., Wooley K.L. (2017). Degradable polyphosphoester-based silver-loaded nanoparticles as therapeutics for bacterial lung infections. Nanoscale.

[38] Ju P.F., Hu J., Li F., Cao Y.W., Li L., Shi D.J., Hao Y., Zhang M.Z., He J.L., Ni P.H. (2018). A biodegradable polyphosphoester-functionalized poly(disulfide) nanocarrier for reduction-triggered intracellular drug delivery. J. Mater. Chem. B.

[39] Ding Q.H., Chen S.Y., Hua W.W., Yoo J., Yoon C., Li Z.Q., Zhao E.G., Kim J.S., Gu M.J. (2025). Photoactivated nanovaccines. Chem. Soc. Rev..

[40] Su M.R., Wang J.K., Zhao N.N., Yu B.R., Wang Y.G., Xu F.J. (2024). Genetically light-enhanced immunotherapy mediated by a fluorinated reduction-sensitive delivery system. Biomaterials.

[41] Deng W., McKelvey K.J., Guller A., Fayzullin A., Campbell J.M., Clement S., Habibalahi A., Wargocka Z., Liang L., Shen C. (2020). Application of mitochondrially targeted nanoconstructs to neoadjuvant X-ray-induced photodynamic therapy for rectal cancer. ACS Cent. Sci..

[42] Clement S., Chen W.J., Deng W., Goldys E.M. (2018). X-ray radiation-induced and targeted photodynamic therapy with folic acid-conjugated biodegradable nanoconstructs. Int. J. Nanomed..

[43] Sun R., Xiang J.J., Zhou Q., Piao Y., Tang J.B., Shao S.Q., Zhou Z.X., Bae Y.H., Shen Y.Q. (2022). The tumor EPR effect for cancer drug delivery: Current status, limitations, and alternatives. Adv. Drug Deliv. Rev..

[44] Zhang B.B., Xu C.F., Sun C.Y., Yu C.S. (2019). Polyphosphoester-Based Nanocarrier for Combined Radio-Photothermal Therapy of Breast Cancer. ACS Biomater. Sci. Eng..

