# Supplementary material for: Polyphosphoester-Based Nanocarriers for Combined X-Ray-Induced Photodynamic Therapy and Immunotherapy
Source: Pharmaceutics. 2026 Mar 24;18(4):399. doi: 10.3390/pharmaceutics18040399 (PMC13118306; doi:10.3390/pharmaceutics18040399)
Supplement: Supplementary file 1 [file pharmaceutics-18-00399-s001.zip › pharmaceutics-4133391-supplementary.pdf]

Supplementary Material

1 Materials

Cyclic phosphate monomer 2-ethylbutoxy-2-oxo-1, 3, 2-dioxaphospholane (BYP) and diblock amphiphilic copolymer PEG_{113}-*b*-$PBYP_{29}$ (PDI: 1.13, Mw: 14100) were synthesized by a similar method described previously, and BYP was distilled under vacuum twice just before use.[1] Verteporfin and R837 were obtained from Aladdin Reagent Co. Ltd. (Shanghai, China). 3-(4,5-dimethylthiazol-2-yl)-2,5-diphenyltetrazolium bromide (MTT) was purchased from Sigma-Aldrich Chemical Co., Ltd. (USA). Dulbecco's modified Eagle's medium (DMEM) and fetal bovine serum (FBS) were purchased from KeyGen Biotech (Nanjing, China). Other chemicals, unless otherwise specified, were of analytical grade and used as received.

2 Characterization Method

The particle size and zeta potential of the samples were determined in aqueous medium using a Malvern ZS90 dynamic light scattering analyzer equipped with a 633 nm He–Ne laser and a 90° detection angle. Data acquisition and processing were conducted with Malvern Dispersion Technology Software version 5.10. Morphological characterization was carried out using a JEOL 2010 high-resolution transmission electron microscope operated at an accelerating voltage of 200 kV. X-ray irradiation at a dose of 4 Gy was conducted using a linear accelerator (Elekta Infinity, Sweden, 10 MeV, 0.4 Gy/min).

3 Tumor Growth Inhibition of NP_{VR} upon X-ray Irradiation

4T1 xenograft-bearing mice were randomly divided into five groups, each consisting of five mice. When the tumors reached approximately 100 mm^3 in size, the mice received intravenous injections of PBS, free VP+R837, NP_V, NP_R, or NP_{VR}, with R837 administered at a dose of 2.5 mg per kilogram once a week. After a 12-hour period post-injection, the tumor sites were irradiated for 10 minutes with an X-ray at a power density of 4.0 Gy. Tumor volume and body weight were monitored every three days, with tumor volume calculated based on the formula: $0.5 \times \text{length} \times \text{width}^2$. After the treatment, the tumors, hearts, livers, spleens, lungs and kidneys were collected, fixed in 4% paraformaldehyde, and subjected to hematoxylin and eosin (H&E) staining for histopathological examination. On day 22, a secondary 4T1 tumor were inoculated in the contralateral flank of mice treated with NP_{VR}+X-ray, while the age-matched mice inoculated the same amount of 4T1 cancer cells were used as controls.

4 Biocompatibility of NP_{VR}

Female BALB/c mice were administered intravenous injections of PBS, free VP+R837, NP_V, NP_R, or NP_{VR} over the course of seven days. On day 8, the mice were euthanized, and blood samples were collected via cardiac puncture for subsequent biochemical analysis. The blood was then centrifuged at 3000 rpm for 10 minutes to isolate the serum. Serum levels of alanine aminotransferase (ALT), aspartate aminotransferase (AST), blood urea nitrogen (BUN), and creatinine (CRE) were measured using an automated biochemical analyzer to assess liver and kidney function. Furthermore, the heart, liver, spleen, lungs, and kidneys from each mouse were harvested for histopathological evaluation. These organs were fixed in 4% paraformaldehyde for 24 hours, embedded in paraffin, sectioned at 6 μm, and stained with hematoxylin and eosin (H&E).

5 Statistical Analysis

The experimental data are expressed as mean ± SD, and statistical analysis was performed using GraphPad Prism 8.0 software for analysis and plotting. Differences between two groups were analyzed using a t-test, while differences among multiple groups were analyzed using one-way ANOVA. Statistical significance was set at $*p < 0.05$.

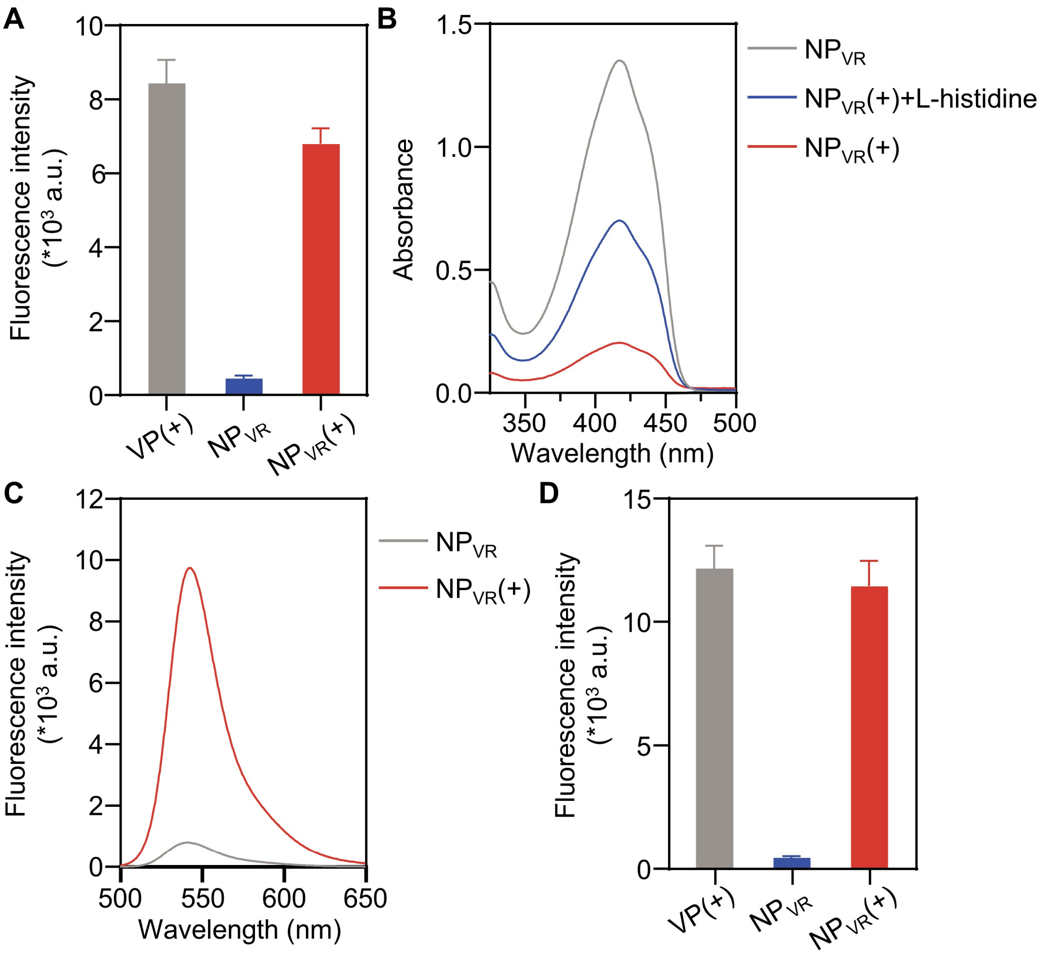

Figure S1. (A) Relative DCF fluorescence intensity at 525 nm in different groups. (B) UV–vis spectra of DPBF in different groups. (C) Fluorescence spectra of dihydrorhodamine 123 in different groups. (D) Relative fluorescence intensity of hydroxyphenyl fluorescein at 515 nm in different groups. (+): X-ray irradiation.

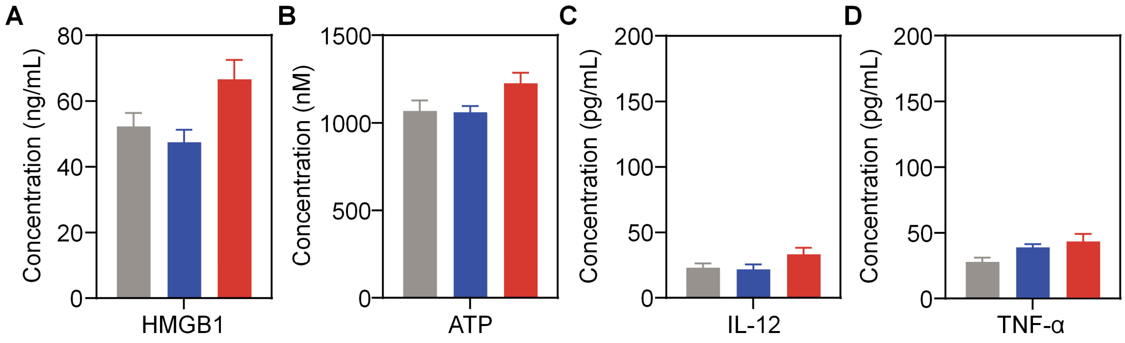

Figure S2. HMGB1 (A) and ATP (B) content of 4T1 cells incubated with NP. IL-12 (C) and TNF-α (D) content in supernatant of DCs after treated with NP.

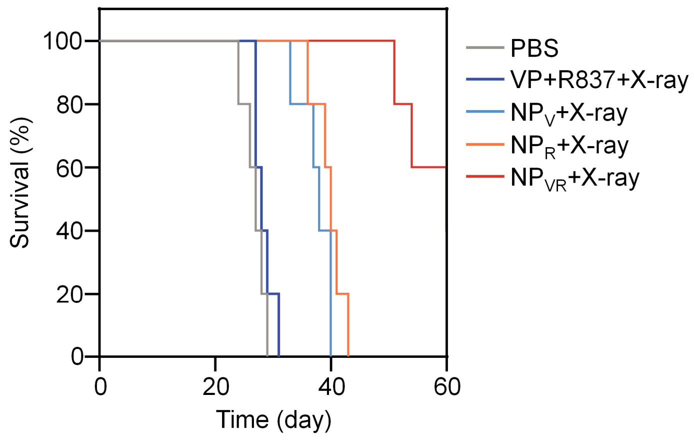

Figure S3. Survival of mice after treatment with various formulations.

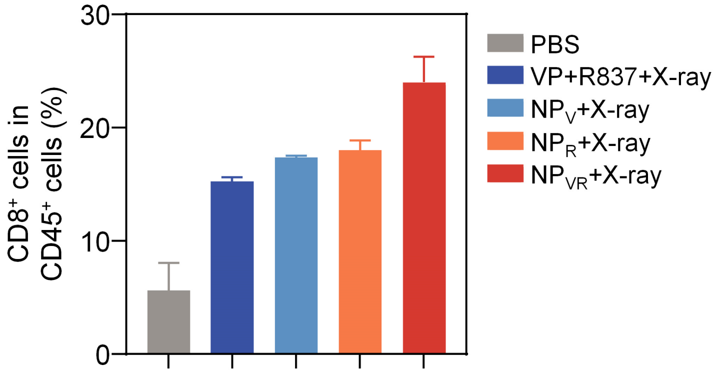

Figure S4. $CD8^+$ T cells in tumors after the treatments.

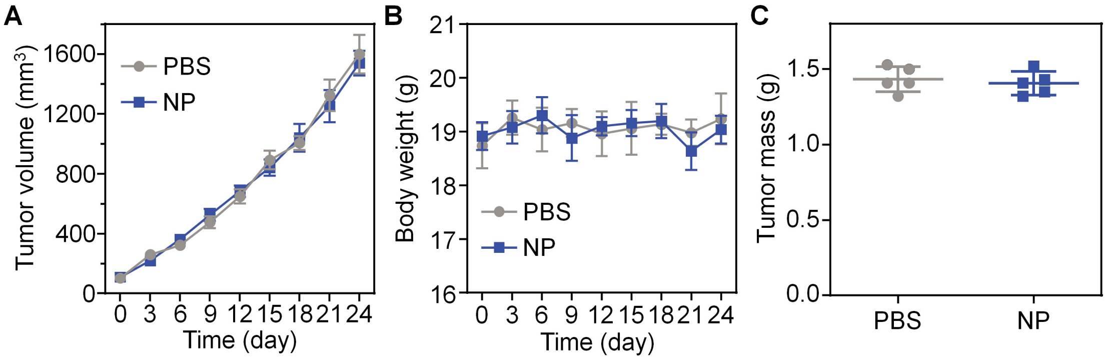

Figure S5. (A) 4T1 tumor volume in different groups during the whole therapeutic period. (B) Monitor of body weight of 4T1 tumor-bearing mice received treatment with PBS or NP. (C) Mass of tumor tissues after the treatment.

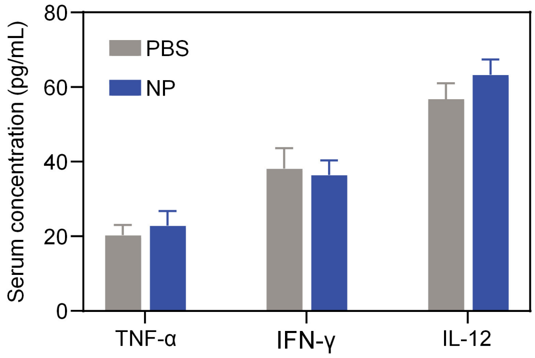

Figure S6. TNF-α, INF-γ and IL-12 content in serum of mice treated with PBS or NP.

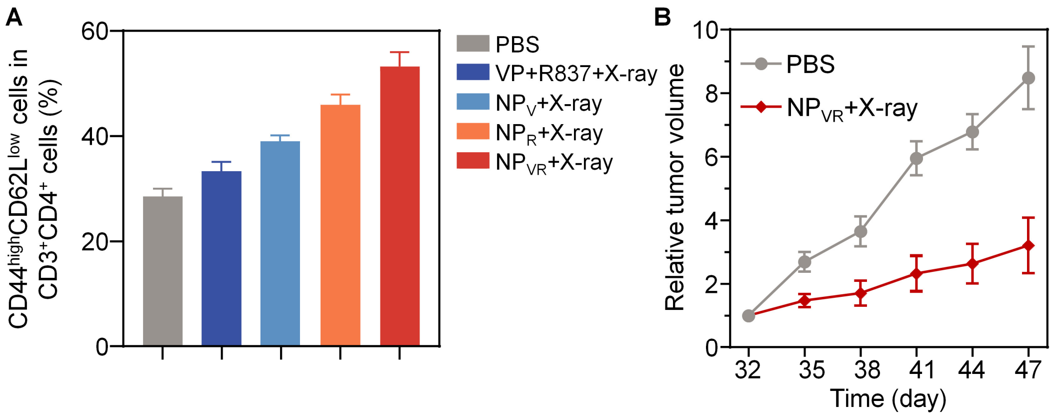

Figure S7. (A) $CD4^+$ memory T cells ($CD3^+CD4^+CD44^{high}CD62L^{low}$) in spleen of mice treated with various formulations. (B) Relative 4T1 tumor growth after the tumor rechallenges.

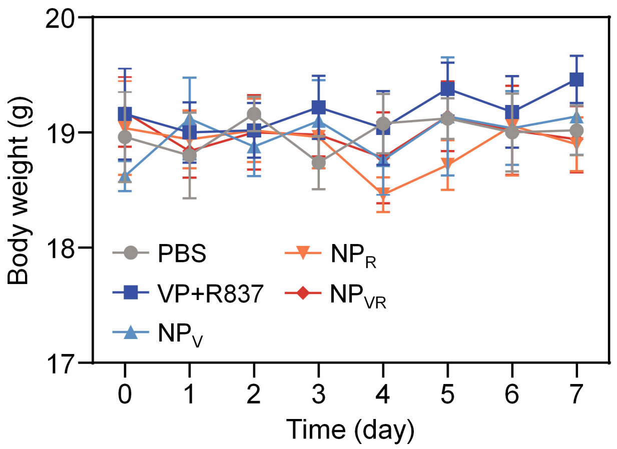

Figure S8. Body weight change of mice after treatment with various formulations.

References

1. Zhang, B.B.; Xu, C.F.; Sun, C.Y.; Yu, C.S. Polyphosphoester-Based Nanocarrier for Combined Radio-Photothermal Therapy of Breast Cancer. *ACS Biomater. Sci. Eng.* **2019**, *5*, 1868-1877. doi: 10.1021/acsbiomaterials.9b00051.
